# Utilization of Proximate Healthcare Facilities and Children’s Wait Times in Senegal: An IV-Tobit Analysis

**DOI:** 10.3390/ijerph20217016

**Published:** 2023-11-03

**Authors:** Abayomi Samuel Oyekale

**Affiliations:** Department of Agricultural Economics and Extension, North-West University Mafikeng Campus, Mmabatho 2735, South Africa; abayomi.oyekale@nwu.ac.za

**Keywords:** healthcare services, universal health coverage, child’s health, treatment waiting time, sick children, Senegal

## Abstract

Universal health coverage (UHC) defines individuals’ timely access to healthcare services without suffering any health-related financial constraints. The Senegalese government has shown commitments towards achievement of UHC as a way of improving access by the population to quality healthcare services. This is very pertinent for promoting some indicators of under-five health in Senegal. Therefore, this study analyzed the factors influencing sick children’s utilization of the nearest healthcare facilities and their wait times in Senegal. The data were from the Service Provision Assessment (SPA) survey, which was conducted in 2018. The instrumental Tobit regression model was used for data analysis. The results showed that 63.50% and 86.01% of the children utilized health posts and publicly owned facilities, respectively. Also, 98.46% of the children utilized urban facilities. The nearest facilities were utilized by 74.55%, and 78.19% spent less than an hour in the facilities. The likelihood of using the nearest healthcare facilities significantly reduced (*p* < 0.05) with caregivers’ primary education, higher education, residence in some regions (Fatick, Kaokack, Saint Louis, Sediou, and Tambacounda), and use of private/NGO not-for-profit facilities, but increased with not having visited any other providers, residence in the Kaffrie region, vomiting symptoms, use of health centers, and use of health posts. Moreover, treatment wait times significantly increased (*p* < 0.05) with the use of nearest facilities, residence in some regions (Diourbel, Kaokack, Matam and Saint Louis), use of private for-profit facilities, use of private not-for-profit facilities, and urban residence, but decreased with secondary education, use of health centers, use of health posts, vomiting symptoms, and showing other symptoms. It was concluded that reduction in wait times and utilization of the nearest healthcare facilities are fundamental to achieving UHC in Senegal. Therefore, more efforts should be integrated at promoting regional and sectoral equities through facilitated public and private healthcare investment.

## 1. Introduction

Timely access to effective healthcare services is a fundamental right of the people. This has been unequivocally emphasized as target 3.8 of the third Sustainable Development Goal (SDG), with associated indicator 3.8.1 emphasizing proper “coverage of essential health services” [1,2]. Universal health coverage (UHC) “ensures everyone has access to effective healthcare services regardless of economic status, race, color, or location” [3,4]. Therefore, the basic understanding of the rights-based approach to health reemphasizes the need to prioritize health policies and their associated interventions in a manner that delivers basic healthcare services in a dignified environment, with utmost equity and efficiency [1]. However, with approximately half of the world’s population being deprived of basic healthcare services, and catastrophic healthcare expenditures subjecting about 100 million people to extreme poverty [5], it cannot be said that enough had been undertaken in attaining UHC. Some statistics have revealed that about one billion people in developing countries, largely from Asia and Sub-Saharan Africa (SSA), are attending healthcare facilities that either lack or have unreliable electricity supply [3]. The concomitant implication of this is that although construction of healthcare facilities gives some indications of numeric progress in the attainment of UHC, attention should be given to availability of essential facilities for the promotion of efficient delivery of medical services. 

It has been emphasized that while UHC is essential in achieving some other targets within the third SDG, such as reduction in maternal and child mortality [4,5,6,7], it will also facilitate achievement of some other SDGs. The Senegalese government had implemented some policy initiatives over the past few decades as a collective demonstration of stringent commitment to the attainment of UHC [8]. Consequently, in 2017, the service coverage index (which measures the percentage of people with adequate access to healthcare services) and incidence of catastrophic expenditure (which depicts medical expenses that pose significant threat to households’ financial capability to meet its basic subsistence expenditures) were 45.4% and 3.3 percent, respectively [9]. The service coverage index can be compared to those of other African countries like Benin, Cameroon, Côte d’Ivoire, and Mauritania with 40%, 46%, 47%, and 41%, respectively. However, Senegal performed much better than these countries in terms of exposure to catastrophic healthcare financing, given that Benin, Cameroon, Côte d’Ivoire, and Mauritania had 10.9%, 10.8%, 12.4%, and 11.7%, respectively. More importantly, the Senegalese government promoted access to healthcare services in some previously underserved areas, while subscription to health insurance increased to 45.39% in 2019 with concurrent reduction in people’s dependence on out-of-pocket payment for medical expenses [10]. The dividends of the different public health interventions in Senegal are clearly reflected in some children’s health development indicators. Specifically, the country is among the few African countries with a drastic reduction in the incidences of stunting among under-five children, which has declined from 34.4% in 1991 to 19.9% in 2019 [11]. 

More importantly, the healthcare system in Senegal withstood the pressure of the COVID-19 pandemic in 2020 and 2021 [12]. With financing being one of the major pillars of healthcare delivery, the Senegalese government’s initiative in using several insurance pathways to achieve UHC has been commended [12,13]. Since community-based health insurance (CBHI) is suffering some fundamental challenges that impact its national acceptability [14,15,16], an innovative health financing mechanism tagged Unité Départementale d’Assurance Maladie (UDAM) has evolved [12]. The implementation and management approaches of UDAM have shown resilience to income shock such as COVID-19, thereby increasing its acceptability [12].

Another important component of quality healthcare delivery in an effort towards UHC is the patients’ wait times [17,18]. This is so because the length of time a patient waits for healthcare service is recognized by the World Health Organization (WHO) as a measure of the responsiveness of the health system [19]. Although the Senegalese government is working towards UHC, long treatment wait times will reduce the satisfaction of patients [16,17,18] and influence compliance with prescribed treatments and the need for follow-up visitations [19,20]. Since a healthcare service delivery system is expected to serve patients in a timely and efficient manner, it is important to understand the factors influencing utilization of nearest healthcare services for the treatment of children and its influence on treatment wait times. In some previous studies that were conducted among adult patients, some socioeconomic factors [21], such as a low level of education [22,23,24], low-income status [22,23,24], employment status [24], age [25], and belonging to a minority group [26], were found to be associated with patients’ treatment wait times. In another study that was carried out among children in need of emergency medical attention, treatment wait times were not significantly influenced by any socioeconomic factors [27]. However, this finding was contrary to those in other studies where some racial or ethnic variables showed significant association with treatment wait times [28,29]. Some other studies analyzed the healthcare facilities’ factors influencing medical treatment wait times and found that first antenatal consultation wait times were lower for rural clinics, while outpatient consultations were lower significantly for the smallest health facilities [30] and public clinics [31].

Provision of timely service is directly anchored on UHC and a precursor of patients’ satisfaction [19,32,33,34,35,36]. Therefore, a proper understanding of healthcare facilities and patient factors influencing wait times at healthcare facilities is of significant relevance to the proper design of interventions to achieve UHC. Therefore, the goal of establishing healthcare facilities will be defeated if available services and the mechanisms of service delivery are not properly aligned to facilitate patients’ satisfaction. This aspect of research has not been fully studied in Senegal using nationally representative datasets. Therefore, this paper is contributing to the existing body of knowledge by utilizing one of the most recent datasets and an appropriate econometric model to analyze the effect of children’s utilization of nearest healthcare facilities on children’s healthcare treatment wait times in Senegal.

## 2. Materials and Methods

In this section, the nature of the data for the study and the methods of their collection are discussed. The description of the selected econometric model for data analysis is also provided.

### 2.1. Data Collection

This study utilized the Senegal’s Service Provision Assessments (SPA) data for 2018. The survey was organized and implemented by the Agence Nationale de la Statistique et de la Démographie (ANSD). The data covered public and private healthcare facilities from the fourteen regions of the country, with representativeness ensured across the different healthcare types, be it hospitals, health centers, health posts, or health huts. The respondents were the healthcare administrators and service providers who provided information for the healthcare inventory and health personnel questionnaires. However, completion of the exit questionnaires for women who attended prenatal clinics and under-five sick children was carried out by the enumerators who also observed the consultation procedures [37].

The survey was based on the sampling frame of the Ministère de la Santé et de l’Action Sociale (MSAS), which included 2092 active health facilities comprising 80 hospitals, 153 health centers, and 1859 health posts. Also, these healthcare facilities comprised 1284 public and 808 private structures. Out of the 2092 healthcare facilities, 378 were selected—36 hospitals, 64 health centers, and 278 health posts—using a stratified sampling procedure. Stratification of the health facilities was performed based on the type and sector of location. Due to their relative importance in healthcare service delivery, 50% of hospitals and health centers were sampled, while between 15–20% of health posts was sampled. Also, out of the 1875 health huts, a sample of 88 was drawn. These facilities were randomly drawn from the selected health posts that have health huts [37].

However, some facilities refused to participate, and only 29 hospitals, 62 health centers, 248 health posts, and 77 health huts were successfully interviewed. Some of the under-five children and women attending these facilities (except health huts) were also interviewed, and this study is based on the data files for under-five children. The data were collected between 15 April and 31 December 2018 by enumerators who were divided into teams. Each team was made up of three people, one of whom served as the leader who ensured that administered questionnaires were in proper order. Healthcare facilities in urban and rural areas were covered. Urban area is defined as that with a minimum of 1000 persons per square kilometer, while rural area has a lesser number [37].

### 2.2. Participation Consent and Ethical Compliance

The healthcare facilities and clients who participated in this survey consented verbally and by signing a portion of the questionnaire before the interviews. More importantly, like previous SPA surveys, the 2018 Service Provision Assessment (SPA) data were collected by Senegal’s National Statistics Agency in conjunction with the Ministère de la Santé et de l’Action Sociale (MSAS). The survey was ethically approved by the Senegal’s Comité National d’Ethique pour Ia Recherche en Santé (CNERS) and the Institutional Review Board of the ICF [38,39].

### 2.3. Limitation of the Study

The fact that the enumerators observed the consultation procedures for each of the sick under-five children may have biased healthcare service providers’ compliance with essential guidelines in treatment administration and prescriptions.

### 2.4. Analytical Model3

This paper utilized the instrumental Tobit regression model for data analysis. The Tobit model was used with left censoring of the data at the minimum because some respondents recorded zero wait times. Tobit regression is the ideal model for analysis of a continuous dependent variable with censoring at certain data points [40]. Therefore, it was assumed that the error term is correlated with utilization of the nearest healthcare facilities, thereby presenting an endogeneity concern [40]. Proper correction of endogeneity is essential to ensure unbiasedness and consistency of estimated parameters. The standard Tobit model can be specified by defining the correlates of unobserved latent variable of wait times ($Y_{i}^{*})$ as

(1) $$y_{1i}^{*}=y_{2i}\beta +x_{1i}\gamma +u_{i}Y_{i}^{*}={\alpha_{1}+\beta }_{k}\sum_{k=1}^{z}X_{ki}+\gamma N_{i}+u_{i}$$

The dependent variable $Y_{i}^{*}$ was left censored at the minimum point of zero (0). $N_{i}$ is the utilization of the nearest healthcare services, which was assumed to be endogenous. $X_{ik}$ is a matrix of other included exogenous variables, and *z* is the number of explanatory variables. $\alpha_{1}$, $\beta_{k}$, and γ are the estimated parameters, and $u_{i}$ is the error term. Table 1 shows the coding formats of the explanatory variables. The reduced form of the correlates of the endogenous covariate is presented as

(2) $$N_{i}={+\pi }_{0}{+\pi }_{1l}\sum_{l=1}^{d}x_{li}+{+\pi }_{2s}\sum_{m=1}^{s}I_{mi}+v_{i}$$

$I_{mi}$ is a matrix of the instrumental variable. The basic property of these variables is that they must be correlated with the endogenous regressor but not correlated with the dependent variable [40]. $\pi_{0}$, $\pi_{1l}$, and $\pi_{2s}$ are the estimated parameters.

## 3. Results

### 3.1. Facilities Attended by Sick Children and Waiting Time

Table 2 shows the distribution of healthcare facilities that were attended by sick children across the regions in Senegal. It shows that most of the healthcare facilities were in urban areas. More specifically, only Longa and Thies regions had rural representation with 34.48% and 1.82%, respectively. The table also reveals that hospitals constituted 26.71% of all the healthcare facilities being attended by children in the Dakar region, as against 0.00% in Kaokack, Kedougou, Kolda, and Thies regions. Except in the Louga region, where health centers accounted for the highest proportion (55.17%) of the healthcare facilities that were attended by sick children, the majority of the children in other provinces attended health posts. The majority of the healthcare facilities were publicly owned by the government, and this is followed by those not-for-profit facilities that were owned by private entities or non-governmental organizations (NGOs).

Table 3 shows the distribution of sick children’s healthcare wait times at the chosen healthcare facilities. It reveals that 53.85% of the sick children waited for less than 30 min, with an average wait time of 14.77 min. Moreover, those who waited 30 < 60 min accounted for 24.34% and had average wait times of 37.43 min. The results also showed that 63.50% of the children were attended to at health posts. This is followed by 25.81% for health centers and 11.19% for hospitals. Similarly, 67.27% of those who waited less than 30 min attended health posts. However, while the percentages of sick children who waited longer hours gradually increased among those who attended hospitals, these percentages gradually decreased among those who chose health posts.

### 3.2. Children’s Utilization of the Nearest Healthcare Facilities

**Figure 1:**
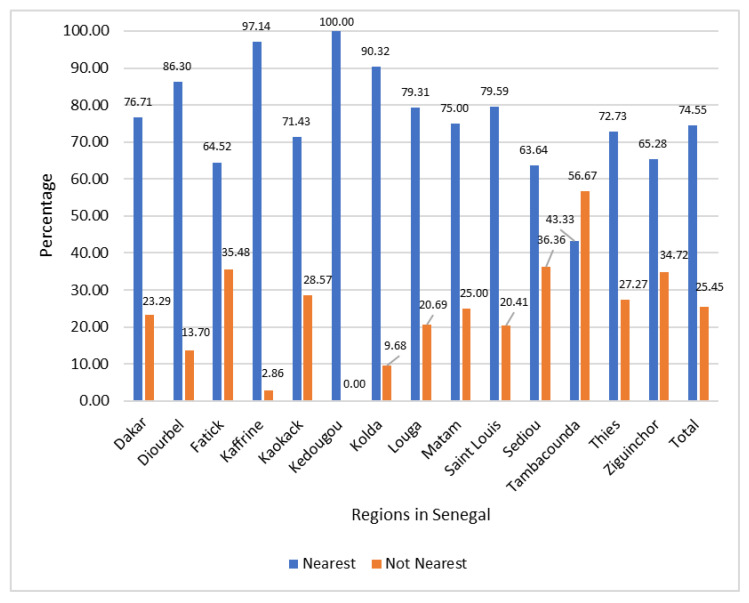
Utilization of the Nearest Healthcare Facilities by Sick Children.

In this study, the nearest healthcare implies facilities that are closest to where the child resided. Figure 1 shows the regional distribution of sick children according to their utilization of nearest healthcare facilities. It reveals that all the children from the Kedougou region and the majority of those from Kaffrine and Kolda utilized the nearest healthcare facilities. However, the Tambacounda, Sediou, Fatick, and Ziguinchor regions recorded the highest percentages for those who were not utilizing nearest healthcare facilities. The results further revealed that about three-quarters of the total children utilized the nearest healthcare facilities.

**Figure 2:**
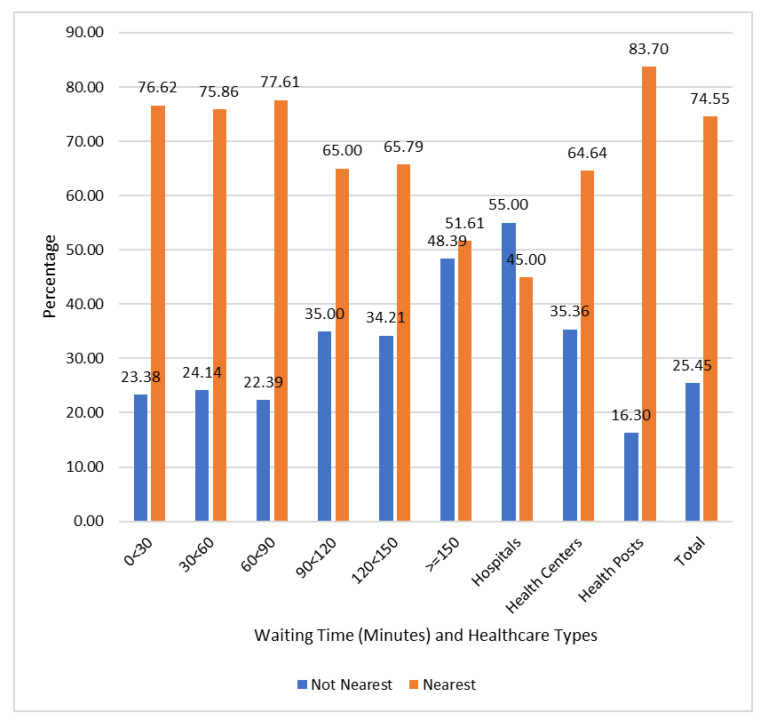
Utilization of Nearest Healthcare Facilities Based on Waiting Time and the Type.

Figure 2 further reveals the distribution of sick children’s utilization of nearest healthcare facilities based on their wait times and the type of facilities. It shows that 51.61% of the children who spent 150 or more minutes before seeing doctors utilized the nearest facilities. Also, 76.62% of the sick children who spent less than 30 min before seeing the doctors utilized the nearest facilities. In addition, 55.00% of the sick children utilized the non-nearest hospitals, while 83.70% utilized the nearest health posts.

**Figure 3:**
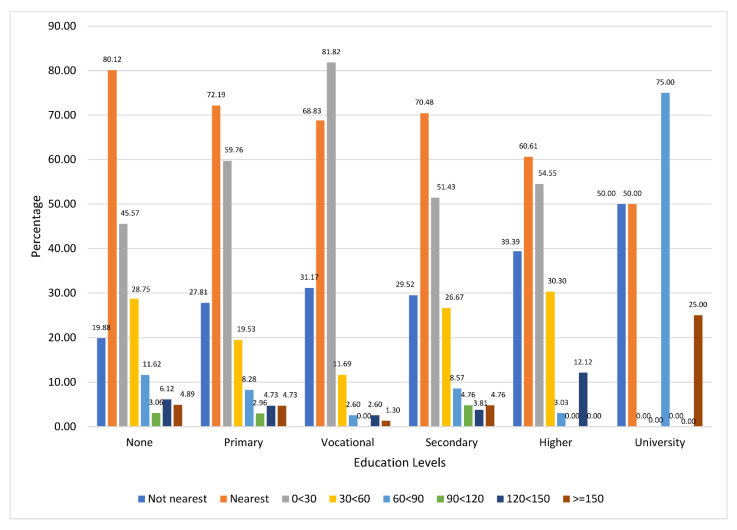
Utilization of Nearest Healthcare Facilities and Waiting Across caregivers’ Education Levels.

Figure 3 shows the distribution of the caregivers’ utilization of the nearest facilities and their wait times across the educational levels. It reveals that out of those without any form of formal education, 80.12% used the nearest facilities, as against 72.19% for those with a primary education. Utilization of the nearest facilities was lowest among those with university education (50.00%). Based on the treatment wait times, the majority of the caregivers with a vocational education (81.82%), primary education (59.76%) and secondary education (51.43%) waited for the least time (0 < 30 min) before being attended. Waiting time of 30 < 60 min was reported by 28.75% of those with no formal education, 26.67% of those with a secondary education, and 30.30% of those with a higher education. Majority of the respondents with university education (75.00%) waited for 60 < 90 min before their sick children were attended to.

### 3.3. Determinants of Sick Children’s Utilization of the Nearest Healthcare Facilities

Table 4 presents the results of the logistic regression, which estimated the determinants of children’s utilization of the nearest healthcare facilities. The parameter of the Wald chi-square is statistically significant (*p* < 0.01). This implies that the model produced a good fit for the data, and the estimated parameters are not jointly statistically equal to zero. The results in Table 3 revealed that among the included demographic variables, the parameters of primary education (*p* < 0.05), higher education (*p* < 0.05), and age of the respondents (*p* < 0.10) showed statistical significance. Precisely, in comparison with those without formal education, the respondents with a primary and higher education, respectively, had 57.73% and 76.24% less likelihood of utilizing the nearest healthcare facilities for the treatment of their sick children. In addition, as the age of the caregivers increased by one year, there was a 1.80% less likelihood of using the nearest healthcare facilities for the child’s treatment.

Moreover, among the estimated regional parameters, those for Diourbel (*p* < 0.10), Fatick (*p* < 0.05), Kaffrine (*p* < 0.05), Kaokack (*p* < 0.01), Matam (*p* < 0.05), Saint Louis (*p* < 0.10), Sediou (*p* < 0.05), and Tambacounda (*p* < 0.01) were statistically significant. The estimated odds ratios revealed that when compared with those from the Dakar region, sick children from the Kaffrine region had 1241.41% higher likelihood of using the nearest healthcare facilities. However, when compared with those from the Dakar region, the children from the Diourbel, Fatick, Kaokack, Matam, Saint Louis, Sediou, and Tambacounda regions, respectively, had 73.00%, 83.18%, 92.75%, 85.50%, 67.64%, 82.62%, and 86.26% less likelihood of using the nearest healthcare facilities.

The results further showed that compared to those who visited other units within the same healthcare facilities, those who did not visit any healthcare facilities had a 162.27% higher likelihood (*p* < 0.05) of using the nearest healthcare facilities. In addition, based on the type of healthcare facilities, when compared with those who used hospitals, the children who utilized health centers (*p* < 0.05) and health posts (*p* < 0.01), respectively, had their likelihood of using the nearest healthcare facilities being significantly higher by 213.48% and 1668.21%, respectively. Also, based on the healthcare management and when compared with those healthcare facilities that were managed by the government, the children who attended private/NGO not-for-profit facilities had their likelihood of using the nearest healthcare facilities significantly lower by 76.96% (*p* < 0.01).

Based on the symptoms that were shown by the children, the children who vomited all that they ate or drank had their likelihood of using the nearest healthcare facilities significantly higher by 578.22% (*p* < 0.01). Also, the children who showed other symptoms rather than vomiting had their likelihood of using the nearest healthcare facilities significantly higher by 337.33% (*p* < 0.05). In addition, the children who showed symptoms of dehydration had their likelihood of using the nearest healthcare facilities significantly higher by 105.40% (*p* < 0.05).

### 3.4. Determinants of Child’s Healthcare Utilization Waiting Time

Table 5 shows the results of the instrumental variable Tobit regression. It reveals that the Wald chi-square statistic is significant (*p* < 0.01), implying that the model produced a good fit for the data, and the estimated parameters are not jointly equal to zero (0). It also signifies that the model produced a good fit of the data. In addition, the Wald test of exogeneity parameter is statistically significant (*p* < 0.05). This is a confirmation of the endogeneity suspicion of the choice of the nearest healthcare facilities variable. Without correcting this problem, estimated parameters will be inconsistent. The results revealed that the parameter of the nearest healthcare facilities is statistically significant (*p* < 0.05). This indicates that other variables held constant; the respondents who utilized the nearest healthcare facilities waited for an average of 114.91 min more than those who did not use the nearest facilities. Moreover, the parameter of urban residence is statistically significant (*p* < 0.01). The result implies that the children from urban areas waited for 90.71 min more than their rural counterparts.

Table 4 further shows that the parameter of secondary education is statistically significant (*p* < 0.05). The result implies that when compared with those without formal education, caregivers with secondary education waited 22.45 min less than those without any formal education. Among the regional variables, the parameters for Diourbel, Kaokack, Matam, and Saint Louis are with a positive sign and statistically significant (*p* < 0.05). The results indicated that compared with those children from Dakar, those from the Dioubel, Kaokack, Matam, and Saint Louis regions had their wait times higher by 19.84, 71.87, 43.93, and 55.68 min, respectively.

Based on the form of hospital management, the children who attended private/NGO not-for-profit facilities had their wait times significantly higher (*p* < 0.01) by 40.78 min when compared with those who used publicly managed healthcare facilities. In a like manner, the children who attended private for-profit facilities had their wait times higher by 32.15 min, when compared with those children who attended publicly managed healthcare facilities. Finally, among the variables that captured the major symptoms shown by the children, the parameters of child vomited everything and showed other symptoms are statistically significant (*p* < 0.05). The results also imply that compared with those children who could not drink or breastfeed, those who vomited everything they ate and showed other symptoms had their wait times reduced by 80.54 and 71.12 min, respectively.

## 4. Discussion

### 4.1. Healthcare Proximity and Types

The results showed that the majority of the children used public healthcare facilities. Utilization of public healthcare facilities is often influenced by proximity and access [41,42]. Moreover, other factors such as attitudes of service providers, the cost and efficiency of medical services, and users’ demographic characteristics are of fundamental relevance [43,44,45,46]. It was also found that the majority of the children utilized health posts. This is consistent with the hierarchical structure of healthcare service delivery in Senegal, with healthcare and referral progressions moving from health posts to health centers and then to hospitals [43]. Healthcare administration is also divided into the central level under the control of the Ministry of Health and Social Affairs (MoHSA), intermediate level under the control of the fourteen regions, and peripheral level under the control of the seventy-seven health districts [47,48].

The results further showed that caregivers mostly chose the nearest facilities for the treatment of their sick children. However, those respondents who utilized the nearest healthcare facilities had significantly higher wait times. More importantly, the use of the nearest healthcare facilities did not imply lesser wait times due to differences in the length of patients’ queue. Therefore, the first impression of patients in a facility based on wait times can determine whether they would come back or not [49].

Furthermore, based on the types of healthcare facility, the caregivers who used health centers and health posts had a higher likelihood of using the nearest healthcare facilities and spending lesser time before treatment. These results are expected because health centers and health posts are the closest facilities to the majority of people. This is due to the health infrastructure decentralization approach that the Senegalese government adopted over the past few decades. Specifically, since the mid-1990s, the government prioritized access of the districts and communities to healthcare facilities, with a more equitable distribution of health centers, health posts, and health huts [50]. This feat had been made possible through the Community Health Programme I (CHP I), Community Health Programme II (CHP II), and Integrated Service Delivery and Health Behaviours project, which were implemented in 2006–2011, 2011–2016, and 2016–2021, respectively [51,52,53]. Therefore, if the healthcare facilities are well equipped, this decentralization is expected to facilitate service delivery with concomitant reduction in healthcare treatment wait times.

In addition, the caregivers who chose to use private/NGO not-for-profit had a lower likelihood of using the nearest healthcare facilities. However, in comparison with those who chose facilities that were managed by the government, the caregivers who used private/NGO (not-for-profit) and private (for-profit) facilities waited for a longer time. These findings can be explained from the fewness of these facilities since they only account for about 25% of the healthcare facilities in Senegal [54]. Specifically, it has been estimated that there are about 3900 private for-profit facilities in Senegal, with 72% of these located in Dakar [55]. However, the private not-for-profit healthcare facilities, which are more than 150 in number, fill the major healthcare needs in rural and peri-urban areas [56]. Since the cost of medical services in these facilities are highly subsidized, the queue length may be very long, thereby resulting in longer wait times.

### 4.2. Caregivers’ Characteristics and Perception of Sickness’ Seriousness

Moreover, the choice of the nearest healthcare facility was facilitated by some caregivers’ demographic characteristics and regional variables. Specifically, attainment of secondary and higher education reduced the likelihood of utilizing the nearest healthcare facilities, while wait times were also reduced among secondary education holders. Maternal education had been found as one of the critical variables influencing utilization of healthcare services, either for sick children or for maternity services [56,57,58,59,60]. However, education can influence caregivers’ perception of the quality of healthcare facilities [60,61,62,63]. It can also drive access to requisite resources to seek the best and timely healthcare services for a sick child [60,61,62,63]. A previous study emphasized that positive children’s health outcomes have been associated with maternal education [62].

The majority of the healthcare facilities were in urban areas, and caregivers from these areas spent more time in healthcare facilities. Although some regional differences exist in the distribution of healthcare facilities across Senegal, the government’s resolution to achieve UHC remains the major driver of equity in the distribution of healthcare infrastructure and personnel [64]. It had been shown that while rural regions like Fatick and Kedougou have a low number of healthcare workers per 10,000 population, highly urbanized regions like Dakar have a high concentration of healthcare workers and possess many hospitals, health centers, and health posts [54,64]. However, due to high demand, the length of patients’ queues in urban areas can be longer than those in rural areas, thereby promoting longer wait times and a higher likelihood of service dissatisfaction.

The caregivers who did not visit any other healthcare providers had a higher likelihood of using nearest facilities and spending less time. These findings are expected because the use of trial and error in the choice of healthcare facilities for the treatment of a child’s illness can result in some serious complications that can degenerate into some emergencies [65,66]. The underlying notion of caregivers’ perceptions of the seriousness of illnesses’ associated infections can impact decisions to utilize some healthcare services [67,68]. However, reluctance in making orthodox medicine the primary contact point when a child is sick can result from long distances to healthcare facilities, financial constraints, and expected long wait times [69,70].

Caregivers’ perception of the seriousness and severity of a child’s illness is of fundamental relevance in the choice of a healthcare facility [71]. The parents often utilize their past experiences to evaluate the severity of an infection [72,73]. In some instances, medical assistance would only be sought when self-medication using the orthodox or traditional approaches have failed [74,75,76]. It was found that children who vomited all that they ate and those who displayed some other symptoms had less wait times. This is expected because in some health centers, treatment preference is often granted to children showing some symptoms like nausea.

## 5. Conclusions

This study seeks to determine the effect of utilization of nearest healthcare facilities on the wait times for children under-5 in Senegal. It was found that majority of the sick children utilized the nearest healthcare facilities, and this significantly increased the wait times. A proper understanding of the correlates of caregivers’ utilization of nearest healthcare facilities for the treatment of children’s illnesses and wait times is of paramount relevance for health facilities’ utilization and policy reforms. This is particularly important for Senegal, given the resources already committed towards achievement of universal health coverage (UHC) over the past few decades. The findings from this study have shown some salient areas for policy interventions for the promotion of nearest healthcare utilization and reduction in wait times.

Specifically, there is a significant rural–urban dichotomy in the distribution of healthcare facilities. This underscores the need for a more equitable distribution of healthcare facilities for facilitating achievement of UHC in Senegal. Interventions that promote establishment of more health posts in rural areas and provide some incentives to rural health personnel can be explored. In addition, there are regional disparities in the caregivers’ utilization of nearest healthcare facilities and wait times. This underscores the need for a more equitable distribution of healthcare facilities with a focus on balancing regional coverage of healthcare services with provision of adequate infrastructure and requisite management. In addition, public healthcare facilities were mostly utilized by sick children. Therefore, establishment of more health centers, health posts, and health huts by the Senegalese government will reduce wait times and facilitate utilization of the nearest healthcare facilities. This can ease some pressing demand on frontline hospitals, which are to serve as final referral points for some complicated health issues requiring specialists’ attentions.

Not-for-profit facilities in Senegal are essential for bridging the usual rural–urban divide in access to healthcare facilities. The operations of these healthcare facilities should be promoted through international assistance and government collaborations, which can be explored via provision of healthcare equipment and financial assistance. More importantly, strengthening the collaboration between specialists in the frontline hospitals and those in not-for-profit facilities can promote quality service delivery, especially for patients with some complicated health problems. Finally, there is the need to promote health education and awareness among children’s caregivers on the need to seek timely healthcare interventions for sick children. This can be explored through different media programs on local radio and television stations. In addition, rural and urban women can be reached with essential health talks by nurses and other health administrators on the critical diseases among children and their early symptoms. This will promote UHC through timely response to children’s healthcare needs, to avoid unnecessary complications and emergencies. The broad implication will be promotion of children’s health outcomes and reduction in child mortality. In addition, future research on the determinants of caregivers’ choice between private and public healthcare facilities in relation to their consultation fees and wait times can be of relevance towards a holistic policy intervention for addressing UHC in Senegal.

## Figures and Tables

**Table 1:** Coding formats of included explanatory variables.

Nature of Variables	Coding Format	Nature of Variables	Coding Format
First visit	Yes = 1, 0 otherwise	*Facility visitation pattern*	
*Type of facilities*		Visited other units (same facility)	Reference variable
Hospital	Reference variable	Visited other facilities	Yes = 1, 0 otherwise
Health center	Yes = 1, 0 otherwise	Visited traditional healers	Yes = 1, 0 otherwise
Health posts	Yes = 1, 0 otherwise	Visited no other facilities	Yes = 1, 0 otherwise
*Education of caregivers*		*Administrator*	
No formal education	Reference variable	Government	Reference variable
Primary	Yes = 1, 0 otherwise	NGO/private not-for-profit	Yes = 1, 0 otherwise
Post-primary/vocational	Yes = 1, 0 otherwise	Private for-profit	Yes = 1, 0 otherwise
Secondary	Yes = 1, 0 otherwise	*Sector of location*	
Higher	Yes = 1, 0 otherwise	Rural	Reference variable
University	Yes = 1, 0 otherwise	Urban	Yes = 1, 0 otherwise
*Regions*		*Dangerous symptoms*	
Dakar	Reference variable	Unable to drink/breastfeed	Reference variable
Diourbel	Yes = 1, 0 otherwise	Vomited everything	Yes = 1, 0 otherwise
Fatick	Yes = 1, 0 otherwise	Had convulsions	Yes = 1, 0 otherwise
Kaffrine	Yes = 1, 0 otherwise	Showed none of these	Yes = 1, 0 otherwise
Kaokack	Yes = 1, 0 otherwise	*Treatment outcome*	
Kedougou	Yes = 1, 0 otherwise	Sent home	Reference variable
Kolda	Yes = 1, 0 otherwise	Referred to other units	Yes = 1, 0 otherwise
Louga	Yes = 1, 0 otherwise	Admitted to same facility	Yes = 1, 0 otherwise
Matam	Yes = 1, 0 otherwise	Sent to laboratory	Yes = 1, 0 otherwise
Saint Louis	Yes = 1, 0 otherwise	Referred to other facility	Yes = 1, 0 otherwise
Sediou	Yes = 1, 0 otherwise	*Instrumental variables*	
Tambacounda	Yes = 1, 0 otherwise	Child dehydrated	Yes = 1, 0 otherwise
Thies	Yes = 1, 0 otherwise	Age of respondents	Years
Ziguinchor	Yes = 1, 0 otherwise		

**Table 2:** Children’s healthcare facilities based on type, management structure, and location.

	Healthcare Facilities’ Type			Management Structure			Location		Total
Region	Hospital	Health Center	Health Post	Government	Private/NGO	Private for-Profit	Urban	Rural	% of Total
Dakar	26.71	32.88	40.41	88.36	7.53	4.11	100.00	0.00	40.35
Diourbel	6.85	13.70	79.45	87.67	10.96	1.37	100.00	0.00	6.91
Fatick	8.06	12.90	79.03	83.87	16.13	0.00	100.00	0.00	7.37
Kaffrine	14.29	14.29	71.43	80.00	20.00	0.00	100.00	0.00	3.14
Kaokack	0.00	17.14	82.86	85.71	14.29	0.00	100.00	0.00	6.44
Kedougou	0.00	14.29	85.71	85.71	14.29	0.00	100.00	0.00	0.23
Kolda	0.00	22.58	77.42	90.32	6.45	3.23	100.00	0.00	2.55
Louga	13.79	55.17	31.03	100.00	0.00	0.00	65.52	34.48	2.58
Matam	13.89	27.78	58.33	100.00	0.00	0.00	100.00	0.00	2.76
Saint Louis	8.16	20.41	71.43	91.84	8.16	0.00	100.00	0.00	6.26
Sediou	5.45	18.18	76.36	81.82	9.09	9.09	100.00	0.00	3.02
Tambacounda	16.67	30.00	53.33	73.33	26.67	0.00	100.00	0.00	6.60
Thies	0.00	45.45	54.55	85.45	12.73	1.82	98.18	1.82	5.37
Ziguinchor	6.94	22.22	70.83	75.00	16.67	8.33	100.00	0.00	6.42
Total	11.19	25.31	63.50	86.01	11.19	2.80	98.46	1.54	100.00

**Table 3:** Distribution of children’s healthcare wait times across healthcare facilities.

	Hospitals		Health Centers		Health Posts		Total		
Waiting Time (minutes)	Freq	%	Freq	%	Freq	%	Freq.	% of Total	Average Time (minutes)
0 < 30	24	6.23	102	26.49	259	67.27	385	53.85	14.77
30 < 60	17	9.77	41	23.56	116	66.67	174	24.34	37.43
60 < 90	12	17.91	17	25.37	38	56.72	67	9.37	61.45
90 < 120	4	20.00	4	20.00	12	60.00	20	2.80	96.25
120 < 150	11	28.95	9	23.68	18	47.37	38	5.31	121.89
≥150	12	38.71	8	25.81	11	35.48	31	4.34	219.06
Total	80	11.19	181	25.31	454	63.50	715	100.00	41.49

**Table 4:** Logistic regression results of the determinants of nearest healthcare utilization.

Variables	Odds Ratio	Robust Std. Error	z Statistics
*Demographic characteristics*			
Primary education	0.422726 **	0.1482863	−2.45
Post-primary/vocational education	0.5661392	0.2451324	−1.31
Secondary education	2.021041	0.9347564	1.52
Higher education	0.2376322 **	0.1626052	−2.10
University education	0.6057401	0.5977742	−0.51
Urban facility	1.013217	1.24154	0.01
Age of respondent	0.9819595 *	0.0103427	−1.73
Provider gender (male)	1.452582	0.4270995	1.27
*Region of residence*			
Diourbel	0.2700261 *	0.1980297	−1.79
Fatick	0.1681637 **	0.1188563	−2.52
Kaffrine	13.41412 **	15.86089	2.20
Kaokack	0.0724768 ***	0.0508559	−3.74
Kedougou	1	-	-
Kolda	0.5396489	0.5425202	−0.61
Louga	0.4616539	0.4290366	−0.83
Matam	0.1449631 **	0.1139752	−2.46
Saint Louis	0.323617 *	0.2183897	−1.67
Sediou	0.1737755 **	0.1375151	−2.21
Tambacounda	0.1373578 ***	0.0944989	−2.89
Thies	0.4315295	0.2695468	−1.35
Ziguinchor	0.3087374	0.2293771	−1.58
*Visitation to other facilities*			
Visited other healthcare facilities	0.6165949	0.6140672	−0.49
Visited traditional healers	0.9438993	0.7194382	−0.08
Visited no other healthcare facilities	2.622721 ***	0.9559713	2.65
First visit to the facilities	1.611194	0.6841424	1.12
*Type of facilities*			
Health center	3.134816 **	1.533333	2.34
Health post	17.68214 ***	9.579755	5.30
*Hospital management types*			
Private/NGO not-for-profit	0.2304086 ***	0.0914951	−3.70
Private for-profit	0.3044427	0.2222639	−1.63
*Symptoms showed by child*			
Child vomits everything	6.782206 ***	4.906293	2.65
Child had convulsion	0.2633315	0.390746	−0.90
Child showed none of the symptoms	4.373339 **	2.757639	2.34
*Treatment outcome*			
Child admitted to same facility	5.033159 *	4.313638	1.89
Child sent to laboratory	7.466214	10.55678	1.42
Child referred to other external facilities	3.242801	3.042426	1.25
Child dehydrated	2.054019 **	0.6736485	2.19
Child referred to another facility	0.3874237 *	0.2116256	−1.74
Satisfied with the services	1.808603	1.524662	0.70
Respiratory infections	0.8333609	0.2478156	−0.61
Digestive infections	0.6781831	0.2327125	−1.13
Malaria infection	0.263787	0.2552897	−1.38
Fever infection	0.7097675	0.2687985	−0.91
Constant	0.0276054 **	0.0449064	−2.21
*Diagnostic indicators*			
Number of observations	715		
Wald chi-square	116.19 ***		

***—significant at 1%; **—significant at 5%; and *—significant at 10%.

**Table 5:** Instrumental Tobit Regression Results of the Determinants of Child’s Waiting Time.

	Coefficients	Std. Error	z Statistics
Nearest health facility	114.9086 **	55.72138	2.06
First visit (yes = 1, 0 otherwise)	−14.83495	11.94657	−1.24
Visited other facilities			
Visited other healthcare facilities	28.54269	18.58913	1.54
Visited traditional healers	−14.21753	13.61236	−1.04
Visited no one	−21.45428 *	11.34546	−1.89
Education			
Primary education	2.095776	7.841755	0.27
Post-primary/vocational education	4.079921	12.87535	0.32
Secondary education	−26.44592 **	11.41158	−2.32
Higher education	3.099546	22.00376	0.14
University education	9.464092	27.16536	0.35
Regions			
Diourbel region	19.83818 **	9.377548	2.12
Fatick region	8.988693	12.03806	0.75
Kaffrine region	−20.79079 *	11.13523	−1.87
Kaokack region	71.87024 ***	20.70972	3.47
Kedougou region	2.68276	14.21874	0.19
Kolda region	−1.052777	9.775943	−0.11
Louga region	−13.60771	16.77396	−0.81
Matam region	43.92891 ***	15.31938	2.87
Saint Louis region	55.67918 ***	13.59337	4.10
Sediou region	8.225394	13.42222	0.61
Tambacounda region	53.54017 *	28.66531	1.87
Thies region	11.40361	8.401836	1.36
Ziguinchor region	−5.805092	10.75012	−0.54
Facility Type			
Health center	−57.96009 ***	15.14235	−3.83
Health post	−84.69879 ***	24.64849	−3.44
Hospital Management Types			
Private/NGO not-for-profit	40.77879 ***	14.1054	2.89
Private for-profit	32.15476 **	12.60406	2.55
Facility Location			
Urban facility	90.7066 ***	21.56068	4.21
Symptoms Shown By Child			
Child vomits everything	−80.53546 **	34.60835	−2.33
Child had convulsion	3.862035	44.06009	0.09
Child showed none of the symptoms	−71.12222 **	32.56936	−2.18
Treatment Outcome			
Child referred to other units within same facility	−38.93408 *	21.92744	−1.78
Child admitted to same facility	−14.01998	19.0114	−0.74
Child sent to laboratory	−30.06189	33.99239	−0.88
Child referred to other facility	−2.995982	15.52097	−0.19
Constant	100.974 ***	37.6959	2.68
Corr (e.x206nearest,e.x201waitingtime)	−0.7535634	0.1425344	
sd (e.x201waitingtime)	52.67709	14.57313	
sd (e.x206nearest)	0.3151537	0.0298647	
Number of observations	715		
Uncensored	688		
Left-censored	27		
Wald chi^2^(35)	133.72 ***		
Log pseudolikelihood	−3657.4276		
Wald test of exogeneity (corr = 0): chi^2^(1)	8.85 ***		

***—significant at 1%; **—significant at 5%; and *—significant at 10%.

## Data Availability

The data were the Service Provision Assessment (SPA) collected by as part of the Demographic Health Survey (DHS) in Senegal. Special authorization to use the data was received, which does not include sharing in any other repositories.

## References

[1] United Nations Transforming Our World: The 2030 Agenda for Sustainable Development. https://sdgs.un.org/2030agenda.

[2] United Nations Development Programme (UNDP) Goal 3 Good Health and Well-,2Being. https://www.undp.org/sustainable-development-goals/good-health.

[3] World Health Organization (WHO) (2023). Close to One Billion People Globally Are Served by Health-Care Facilities with No Electricity Access or with Unreliable Electricity. https://www.who.int/news/item/14-01-2023-close-to-one-billion-people-globally-are-served-by-health-care-facilities-with-no-electricity-access-or-with-unreliable-electricity.

[4] Howden-Chapman P., Siri J., Chisholm E., Chapman R., Doll C.N., Capon A. (2017). SDG 3: Ensure Healthy Lives and Promote Wellbeing for All at All Ages. A Guide to SDG Interactions: From Science to Implementation.

[5] World Bank and World Health Organization (WHO) (2017). World Bank and WHO: Half the World Lacks Access to Essential Health Services, 100 Million still Pushed into Extreme Poverty Because of Health Expenses. https://www.who.int/news/item/13-12-2017-world-bank-and-who-half-the-world-lacks-access-to-essential-health-services-100-million-still-pushed-into-extreme-poverty-because-of-health-expenses#:~:text=of%20health%20expenses-,World%20Bank%20and%20WHO%3A%20Half%20the%20world%20lacks%20access%20to,poverty%20because%20of%20health%20expenses&text=At%20least%20half%20of%20the,the%20World%20Bank%20and%20WHO.

[6] Sanogo N.D., Fantaye A.W., Yaya S. (2019). Universal health coverage and facilitation of equitable access to care in Africa. Front. Public Health.

[7] Derkyi-Kwarteng A.N., Agyepong I.A., Enyimayew N., Gilson L. (2021). A narrative synthesis review of out-of-pocket payments for health services under insurance regimes: A policy implementation gap hindering universal health coverage in sub-Saharan Africa. Int. J. Health Policy Manag..

[8] République du Sénégal, Ministère de la Santé et de l’Action Sociale (MSAS) (2017). Stratégie Nationale de Financement de la Santé (SNFS) Pour Tendre vers la Couverture Sanitaire Universelle.

[9] World Health Organization (2019). Primary Health Care on the Road to Universal Health Coverage-2019 Monitoring Report.

[10] (2019). Performances Majeures de la CMU [Internet] Agence de la Couverture Maladie Universelle. http://www.agencecmu.sn/performances-majeurs-de-la-cmu.

[11] UNICEF (2023). Child Malnutrition. https://data.unicef.org/topic/nutrition/malnutrition/.

[12] Ridde V., Kane B., Mbow N.B., Senghor I., Faye A. (2022). The resilience of two departmental health insurance units during the COVID-19 pandemic in Senegal. BMJ Glob. Health.

[13] Waelkens M.-P., Werner S., Bart C. (2017). Community Health Insurance in Low- and Middle-Income Countries. International Encyclopedia of Public Health.

[14] Daff B.M., Diouf S., Diop E.S.M., Mano Y., Nakamura R., Sy M.M., Tobe M., Togawa S., Ngom M. (2020). Reforms for financial protection schemes towards universal health coverage, Senegal. Bull. World Health Organ..

[15] Ly M.S., Faye A., Ba M.F. (2022). Impact of community-based health insurance on healthcare utilisation and outof-pocket expenditures for the poor in Senegal. BMJ Open.

[16] Agence Nationale de la Statistique et de la Démographie, ICF (2020). Sénégal: Enquête Démographique et de Santé Continue (EDS-Continue 2019)—Tableaux.

[17] Blanchet K., Gordon I., Gilbert C.E., Wormald R., Awan H. (2012). How to achieve universal coverage of cataract surgical services in developing countries: Lessons from systematic reviews of other services. Ophthalmic Epidemiol..

[18] Morgan R., Ensor T., Waters H. (2016). Performance of private sector health care: Implications for universal health coverage. Lancet.

[19] Sun J., Lin Q., Zhao P., Zhang Q., Xu K., Chen H., Hu C.J., Stuntz M., Li H., Liu Y. (2017). Reducing wait times and raising outpatient satisfaction in a Chinese public tertiary general hospital-an interrupted time series study. BMC Public Health.

[20] Wouters A. (1991). Essential national health research in developing countries: Health care financing and the quality of care. Int. J. Health Plan. Manag..

[21] McPake B. (1993). User charges for health services in developing countries: A review of the economic literature. Soc. Sci. Med..

[22] Gilson L., Alilio M., Heggenhougen K. (1994). Community satisfaction with primary health care services: An evaluation undertaken in the Morogoro region of Tanzania. Soc. Sci. Med..

[23] Aharony L., Strasser S. (1993). Patient satisfaction: What we know about and we still need to explore. Med. Care Rev..

[24] Ware J., Snyder M., Wright W., Davies A. (1983). Defining and measuring patient satisfaction with medical care. Eval. Program Plan..

[25] Landi S., Ivaldi E., Testi A. (2019). Socioeconomic Status and Waiting Times for Health Services: Current Evidences and Next Area of Research. Health Serv. Insights.

[26] Monstad K., Engesæter L.B., Espehaug B. (2014). Waiting time and socioeconomic status—An individual-level analysis. Health Econ..

[27] Laudicella M., Siciliani L., Cookson R. (2012). Waiting times and socioeconomic status: Evidence from England. Soc. Sci. Med..

[28] García-Corchero J.D., Jiménez-Rubio D. (2022). Waiting times in healthcare: Equal treatment for equal need?. Int. J. Equity Health.

[29] McIntyre D., Marschner S., Thiagalingam A., Pryce D., Chow C.K. (2023). Impact of Socio-demographic Characteristics on Time in Outpatient Cardiology Clinics: A Retrospective Analysis. Inquiry.

[30] Gallego G., Dew A., Lincoln M., Bundy A., Chedid R.J., Bulkeley K., Brentnall J., Veitch C. (2017). Access to therapy services for people with disability in rural Australia: A carers’ perspective. Health Soc. Care Community.

[31] Ndu I.K., Osuorah C.D.I., Amadi O.F., Ekwochi U., Ekeh B.C., Nduagubam O.C., Okeke I.B. (2020). Evaluation of Wait Time in the Children’s Emergency and Outpatient Units of a Tertiary Hospital in Southeast Nigeria. J. Emerg. Trauma Shock.

[32] James C.A., Bourgeois F.T., Shannon M.W. (2005). Association of race/ethnicity with emergency department wait times. Pediatrics.

[33] Park C.Y., Lee M.A., Epstein A.J. (2009). Variation in emergency department wait times for children by race/ethnicity and payment source. Health Serv. Res..

[34] Wagenaar B.H., Gimbel S., Hoek R., Pfeiffer J., Michel C., Cuembelo F., Quembo T., Afonso P., Gloyd S., Lambdin B.H. (2016). Wait and consult times for primary healthcare services in central Mozambique: A time-motion study. Glob. Health Action.

[35] Newman R., Gloyd S., Nyangezi J., Machobo F., Muiser J. (1998). Satisfaction with outpatient health care services in Manica Province, Mozambiaue. Health Policy Plan..

[36] Harper P.R., Gamlin H.M. (2003). Reduced outpatient wait times with improved appointment scheduling: A simulation modeling approach. OR Spectr..

[37] Demographic and Health Survey (DHS) Definition of Urban and Rural Areas. https://dhsprogram.com/pubs/pdf/FR01/11AppendixA.pdf.

[38] Demographic and Health Survey (DHS) Protecting the Privacy of DHS Survey Respondents. https://dhsprogram.com/methodology/Protecting-the-Privacy-of-DHS-Survey-Respondents.cfm.

[39] Agence Nationale de la Statistique et de la Démographie (ANSD) et ICF (2020). Sénégal: Enquête Continue sur la Prestation des Services de Soins de Santé (ECPSS) 2018.

[40] Chesher A., Kim D., Rosen A.M., IV Methods for Tobit Models (2023). The Institute for Fiscal Studies Department of Economics, UCL Cemmap Working Paper CWP16/22. https://ifs.org.uk/sites/default/files/2022-10/CWP1622-IV-Methods-for-Tobit-Models.pdf.

[41] Ridde V., Sombie I. (2012). Street-level workers’ criteria for identifying indigents to be exempted from user fees in Burkina Faso. Trop. Med. Int. Health.

[42] Ridde V. (2008). “The problem of the worst-off is dealt with after all other issues”: The equity and health policy implementation gap in Burkina Faso. Soc. Sci. Med..

[43] Al-Ghanim S.A. (2004). Factors influencing the utilisation of public and private primary health care services in Riyadh City. JKAU Econ. Adm..

[44] Awoyemi T.T., Obayelu O.A., Opaluwa H.I. (2011). Effect of distance on utilization of health care services in rural Kogi State, Nigeria. J. Hum. Ecol..

[45] Paul P., Chouhan P. (2020). Socio-demographic factors influencing utilization of maternal health care services in India. Clin. Epidemiol. Glob. Health.

[46] Obiechina G.O., Ekenedo G.O. (2013). Factors affecting utilization of university health services in a tertiary institution in South-West Nigeria. Niger. J. Clin. Pract..

[47] Paul E., Ndiaye Y., Sall F.L., Fecher F., Porignon D. (2020). An assessment of the core capacities of the Senegalese health system to deliver Universal Health Coverage. Health Policy Open.

[48] (2019). République du Sénégal, Ministère de la Santé et de l’Action sociale Plan National de Développement Sanitaire et Social (PNDSS) 2019–2028. http://www.sante.gouv.sn/publications/plan-national-de-développement-sanitaire-et-social-2019-2028.

[49] Masango-Makgobela A.T., Govender I., Ndimande J.V., Ndimande J.V. (2013). Reasons patients leave their nearest healthcare service to attend Karen Park Clinic, Pretoria North. Afr. J. Prim. Health Care Fam. Med..

[50] USAID (2019). Senegal’s Community-Based Health System Model: Structure, Strategies, and Learning. https://www.advancingpartners.org/sites/default/files/technical-briefs/apc_senegal_brief_508.pdf.

[51] Agence Nationale de la Statistique et de la Démographie (ANSD) [Sénégal], and ICF (2018). Sénégal: Enquête Démographique et de Santé Continue (EDS-Continue 2017).

[52] Salif N., Ayad M. (2006). Enquête Démographique et de Santé au Sénégal 2005.

[53] Agence Nationale de la Statistique et de la Démographie (ANSD) [Sénégal], et ICF (2016). Sénégal: Enquête Démographique et de Santé Continue (EDS-Continue 2015).

[54] PHCPI Senegal: Physical Infrastructure. https://www.improvingphc.org/senegal-physical-infrastructure.

[55] Bettina B., Barnes J., Carmona A., Kpangon A., Riley P., Mohebbi E., Miles L. (2016). Senegal Private Health Sector Assessment: Selected Health Products and Services. Strengthening Health Outcomes through the Private Sector Project.

[56] Hong T.K., Dibley M.J., Tuan T. (2003). Factors affecting utilization of health care services by mothers of children ill with diarrhea in rural Vietnam. Southeast Asian J. Trop. Med. Public Health.

[57] Feikin D.R., Nguyen L.M., Adazu K., Ombok M., Audi A., Slutsker L., Lindblade K.A. (2009). The impact of distance of residence from a peripheral health facility on pediatric health utilisation in rural western Kenya. Trop. Med. Int. Health.

[58] Becker S., Peters D.H., Gray R.H., Gultiano C., Black R.E. (1993). The determinants of use of maternal and child health services in Metro Cebu, the Philippines. Health Transit. Rev..

[59] Sreeramareddy C.T., Shankar R.P., Sreekumaran B.V., Subba S.H., Joshi H.S., Ramachandran U. (2006). Care seeking behaviour for childhood illness-a questionnaire survey in western Nepal. BMC Int. Health Hum. Rights.

[60] Black D., Morris J.N., Smith C., Townsend P., Whitehead M. (1988). Inequalities in Health: The Black Report. The Health Divide.

[61] Gage T.B., Fang F., O’Neill E., Dirienzo G. (2013). Maternal education, birth weight, and infant mortality in the United States. Demography.

[62] Prickett K.C., Augustine J.M. (2016). Maternal Education and Investments in Children’s Health. J. Marriage Fam..

[63] Amwonya D., Kigosa N., Kizza J. (2022). Female education and maternal health care utilization: Evidence from Uganda. Reprod. Health.

[64] Nagai M., Fujita N., Diouf I.S., Salla M. (2017). Retention of qualified healthcare workers in rural Senegal: Lessons learned from a qualitative study. Rural Remote Health.

[65] Kahabuka C., Kvale G., Moland K.M., Hinderaker S.G. (2011). Why caretakers bypass primary health care facilities for child care—A case from rural Tanzania. BMC Health Serv. Res..

[66] Federal Ministry of Health Guideline for Implementation of a Patient Referral System. Addis Ababa, Ethiopia 2010 [27/05/2021]..

[67] Oyekale A.S. (2015). Assessment of Malawian Mothers’ Malaria Knowledge, Healthcare Preferences and Timeliness of Seeking Fever Treatments for Children Under Five. Int. J. Environ. Res. Public Health.

[68] Bekele M., Urgessa M., Kumsa K., Sinba E. (2023). Contributing factors of delay in seeking treatment for childhood diarrheal diseases in Berbere Woreda, Ethiopia: An unmatched case–control study. J. Health Popul. Nutr..

[69] Lungu E.A., Biesma R., Chirwa M., Darker C. (2016). Healthcare seeking practices and barriers to accessing under-five child health services in urban slums in Malawi: A qualitative study. BMC Health Serv. Res..

[70] Kassile T., Lokina R., Mujinja P., Mmbando B.P. (2014). Determinants of delay in care seeking among children under five with fever in Dodoma region, central Tanzania: A cross-sectional study. Malar. J..

[71] Abdulraheem I.S., Parakoyi D.B. (2009). Factors affecting mothers’ healthcare-seeking behaviour for childhood illnesses in a rural Nigerian setting. Early Child Dev. Care.

[72] Falade C.O., Ogundiran M.O., Bolaji M.O., Ajayi I.O., Akinboye D.O., Oladepo O., Adeniyi J.D., Oduola A.M. (2005). The Influence of Cultural Perception of Causation, Complications, and Severity of Childhood Malaria on Determinants of Treatment and Preventive Pathways. Int. Q. Community Health Educ..

[73] Williams A., O’Rourke P., Keogh S. (2009). Making choices: Why parents present to the emergency department for non-urgent care. Arch. Dis. Child..

[74] Mensah B.N., Agyemang I.B., Afriyie D.K., Amponsah S.K. (2019). Self-medication practice in Akuse, a rural setting in Ghana. Niger. Postgrad. Med. J..

[75] Asenso-Okyere W.K., Dzator J.A., Osel-akoto I. (1996). The behaviour towards malaria care—A multinomial logit approach. Soc. Indic. Res..

[76] Tabuti J.R., Dhillion S.S., Lye K.A. (2003). Traditional medicine in Bulamogi county, Uganda: Its practitioners, users and viability. J. Ethnopharmacol..

